# Macrogenomes reveal microbial-mediated microplastic degradation pathways in the porcine gut: a hope for solving the environmental challenges of microplastics

**DOI:** 10.3389/fmicb.2024.1442946

**Published:** 2024-07-29

**Authors:** Tao Wang, Yuheng Luo, Bing Yu, Ping Zheng, Zhiqing Huang, Xiangbing Mao, Jie Yu, Junqiu Luo, Hui Yan, Jun He

**Affiliations:** ^1^Institute of Animal Nutrition, Sichuan Agricultural University, Chengdu, China; ^2^Key Laboratory of Animal Disease-Resistant Nutrition, Chengdu, China

**Keywords:** pigs, MP-degrading enzymes, microbial degradation, microplastic, intestinal contents

## Abstract

It is increasingly recognized that microplastics (MPs) are being transmitted through the food chain system, but little is known about the microorganisms involved in MP degradation, functional biodegradation genes, and metabolic pathways of degradation in the intestinal tract of foodborne animals. In this study, we explored the potential flora mainly involved in MP degradation in the intestinal tracts of Taoyuan, Duroc, and Xiangcun pigs by macrogenomics, screened relevant MP degradation genes, and identified key enzymes and their mechanisms. The pig colon was enriched with abundant MP degradation-related genes, and gut microorganisms were their main hosts. The fiber diet did not significantly affect the abundance of MP degradation-related genes but significantly reduced their diversity. We identified a total of 94 functional genes for MP degradation and classified them into 27 categories by substrate type, with polystyrene (PS), polyethylene terephthalate (PET), and di(2-ethylhexyl) phthalate (DEHP) were the most predominant degradation types. The MP degradation functional genes were widely distributed in a variety of bacteria, mainly in the phylum *Firmicutes* and *Bacteroidetes*. Based on the identified functional genes for MP degradation, we proposed a hypothetical degradation mechanism for the three major MP pollutants, namely, PS, PET, and DEHP, which mainly consist of oxidoreductase, hydrolase, transferase, ligase, laccase, and isomerase. The degradation process involves the breakdown of long polymer chains, the oxidation of short-chain oligomers, the conversion of catechols, and the achievement of complete mineralization. Our findings provide insights into the function of MP degradation genes and their host microorganisms in the porcine colon.

## Introduction

With global production of plastics steadily increasing and millions of tons released into the environment each year, plastics have become the most ubiquitous man-made substance on the planet today (Ragossnig and Agamuthu, 2021). As a persistent pollutant, plastics undergo degradation processes including exposure to solar radiation, physical abrasion, and biodegradation. These processes cause large plastic items to break down into smaller particles known as microplastics (MPs), which are defined as plastic fragments and particles less than 5 mm in diameter (Vethaak and Legler, 2021). MPs are prevalent in a wide range of environments, including marine, freshwater, atmospheric, and soil ecosystems (Ragossnig and Agamuthu, 2021). Furthermore, they have been detected in the digestive tracts of animals and in human blood, brain, and placenta, posing significant ecological and human health hazards (Vethaak and Legler, 2021; Dusza et al., 2023; Osman et al., 2023).

As research has progressed, MPs are potentially harmful to the immune system, metabolism, and overall health of animals (da Silva Brito et al., 2022; Li et al., 2022; Wu et al., 2022). Studies have demonstrated that MP particles can enter the bodies of animals, including the digestive tracts of fish, birds, and other wildlife (Chang et al., 2022; Liang et al., 2023). These particles may stimulate an inflammatory response, leading to intestinal inflammation, growth inhibition, and developmental restrictions (Teng et al., 2022). Chemicals in MPs can release toxic substances, such as phthalates and polybrominated biphenyls, which have also been found to disrupt the normal functioning of the endocrine system in animals, affecting metabolic rates and energy balance, leading to changes in body weight, reproductive problems, and other health effects (Zhang et al., 2022). Moreover, MPs may influence animal behavior and ecosystem functions, such as food chain transmission and ecological niche alteration, adversely affecting the stability of entire ecosystems (Ahmad et al., 2024). Although much remains unknown about the effects of MPs on animals, existing research has raised significant concerns. Therefore, to protect wildlife health and ecosystem stability, further in-depth studies are required to investigate the mechanisms by which MPs affect the immunity, metabolism, and overall health of animals. Additionally, implementing protective measures to mitigate the potential harm caused by MPs to animals and the environment is crucial.

China is the world’s largest producer and consumer of pork. MPs have been shown to accumulate in the intestinal tract and blood of foodborne animals through feed and other pathways (Wu et al., 2021; Xu et al., 2022; Xia et al., 2024). Animal gut microbes can degrade various plastic polymers through enzymatic processes (Amobonye et al., 2021). Therefore, understanding the mechanisms of MP degradation by gut microbes is essential to address the problem of MP contamination in foodborne animals. However, the microorganisms involved in MP degradation, the functional genes for biodegradation, and the metabolic pathways of degradation are still poorly understood. It is crucial to explore and understand the genetic potential of microbial enzymes in MP biodegradation. With the rapid development of high-throughput sequencing technology, the metagenomics approach has become a powerful tool for identifying plastic-degrading microorganisms and enzymes (Danso et al., 2019; Wani et al., 2023). Using metagenomics, researchers can study important microbial taxa associated with plastic degradation, discover new plastic-degrading enzymes from uncultured microorganisms, and reveal metabolic pathways associated with MPs. In this study, we aimed to compare MP degradation-related genes and host microorganisms in the intestines of Chinese local pig breeds, commercial pig breeds, and crossbred pig breeds under different fiber level diets using metagenomics. This comparison is vital for our comprehensive understanding and elucidation of the enzymes and pathways that may be involved in MP degradation in the porcine intestinal tract.

## Materials and methods

### Ethics statement

The management of animal experiments involved in the research shall refer to the “Regulations on the Administration of Laboratory Animals” (Ministry of Science and Technology, China, revised in June 2004). Sample collection was approved by the Institutional Animal Care and Use Committee of Sichuan Agricultural University, Sichuan, China (No. 20181105), and operated in strict accordance with ethical guidelines.

### Animal trial and sample collection

The 60-day-old Taoyuan pigs (average weight: 13.87 ± 0.58 kg, purchased from Xiangcun Hi-Tech Agriculture Co., Ltd.), Duroc pigs (average weight: 18.50 ± 1.09 kg, purchased from Linli Tianxin Breeding Co., Ltd.), and their crossbreed Xiangcun pigs (average weight: 14.47 ± 0.15 kg, purchased from Xiangcun Hi-Tech Agriculture Co., Ltd.) were selected in the experiment, and a 3 × 2 feeding management model and a 3 × 2 design of variance were used. The 3 × 2 feeding model and 3 × 2 design of variance were used, i.e., the three pig breeds were divided into six groups (DL: Duroc low-fiber diet group, *n* = 10; DH: Duroc high-fiber diet group, *n* = 10; XL: Xiangcun pig low-fiber diet group, *n* = 10; XH: Xiangcun pig high-fiber diet group, *n* = 10; TL: Taoyuan pig low-fiber diet group, *n* = 10; XH: Taoyuan pig high-fiber diet group, *n* = 10) and were fed high-fiber diets (crude fiber: 6–7%; digestible energy: 3.5%; and crude protein: 19.16%.) and low-fiber diets (crude fiber: 2–3%; digestible energy: 3.49%; and crude protein: 19.15%), with nutrient levels referenced to the National Research Council (NRC, 2012) standards. Wheat bran fiber was purchased from Chengdu Tubaite Technology Co., Ltd. (manufacturer: JRS, model: WF200, purity >95%). All pigs were housed in single pens with feeders and drinkers in each pen at a room temperature of 28°C and fed *ad libitum*. The experiment lasted for 28 days, and 60 pigs were sampled on the 29th day, and the colonic contents were collected and frozen at −80°C for use in subsequent experiments (Liu et al., 2022; Wang et al., 2023).

### Metagenome sequencing and data analysis

Paired-end libraries were constructed by NEXTFLEX Rapid DNA Seq (Bioo Scientific, Austin, TX, United States) using 1 μg of high-quality DNA constructs, and then sequenced using the Novaseq6000 platform from Shanghai Majorbio BioPharmaceuticals Biotechnology Co Ltd. (Shanghai, China). Raw data were filtered using Trimmomatic v0.38 to remove reads containing more than three ambiguous nucleotides with an average quality score of <20 and to remove artificially duplicated reads. Clean reads were then assembled into contigs using MEGAHIT (Li et al., 2015) (https://github.com/voutcn/megahit, version 1.1.2). Only overlapping clusters of ≥300 bp were retained for subsequent analysis. Open reading frames (ORFs) of all assembled overlap clusters were predicted using MetaGene and translated into amino acid sequences. A catalog of non-redundant genes was constructed using CD-HIT (Fu et al., 2012) (http://www.bioinformatics.org/cd-hit/, version 4.6.1) with 90% sequence identity and 90% coverage. High-quality reads were aligned to the non-redundant gene catalog to calculate gene abundance with 95% identity using SOAPaligner (http://soap.genomics.org.cn/, version 2.21). Sequence data associated with this project have been deposited in the NCBI Short Read Archive database (Accession Number: PRJNA849732).

### Statistics

Data preprocessing was performed using Excel 2019 (Microsoft, United States), and data statistics were performed using SPSS 22.0 (IBM Corp, United States). Graphical display of results was performed using GraphPad Prism 10. RPKM value was used for heat map data, *Z-score* was used for data standardization, and average clustering was used for cluster analysis. Correlation analysis used Spearman. The “protest” function in the vegan package was used to analyze the Procrustes correlation between the bacteriome and the MP degradation genes. Principal coordinate analysis (PCoA) and non-metric multidimensional scaling (NMDS) analyses were performed using the pure vegetation package in R software. The distribution of MP degradation genes in bacteria of different taxonomic levels was plotted as a Sankey diagram using the networkD3 package^1^ in R (v3.6.2). The mulberry graph is drawn using the plotly package in the R software.

Reads per kilobase million (RPKM):


$$RPKM_{i}=\frac{R_{i}*10^{6}}{L_{i}*\sum_{1}^{n}\left(R_{j}\right)}$$

$R_{i}$ represents the abundance value of Genei in a sample, that is, the number of reads aligned to Genei in the sample; $L_{i}$ represents the nucleotide length of Genei; and $\overset{n}{\underset{1}{\sum }}\left(R_{j}\right)$ represents the sum of the reads corresponding to all genes in the sample.

Experimental design using a 3 × 2 design, two-way ANOVA was used to analyze two main effects and interaction effects. The results were expressed as mean ± standard error, and a *p*-value of *<0.05* indicated a significant difference.

## Result

### Differences in dietary and genetically mediated gene profiles for MP degradation in porcine colonic contents

**Figure 1:**
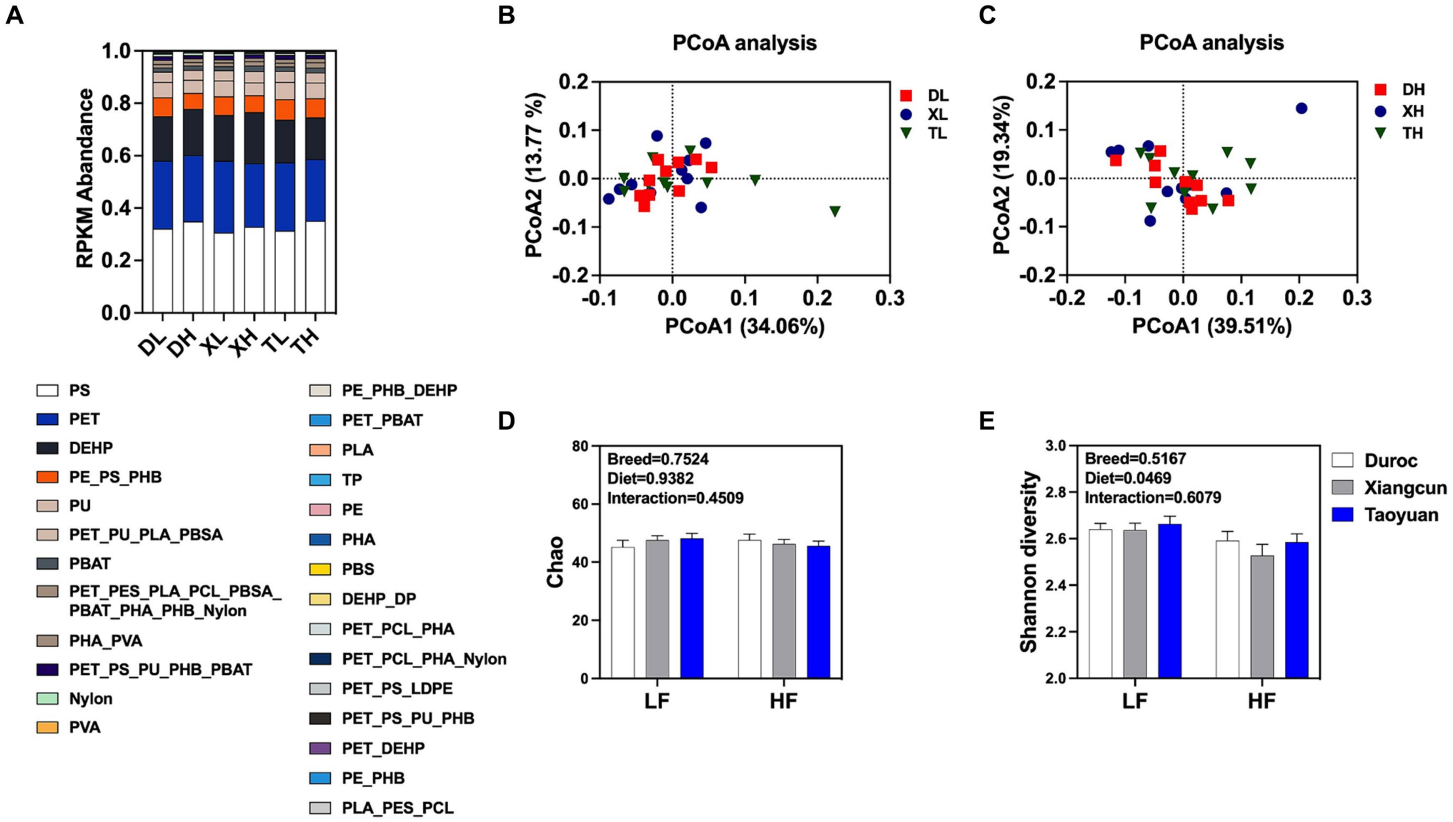
Relative abundance of different types of MP degradation genes **(A)**. Plots of PCoA analysis between different breeds at low fiber level **(B)** and high fiber level **(C)**. Analysis of the alpha diversity of MP degradation genes **(D,E)**. DL, Duroc pig group fed with low fiber level; DH, Duroc pig group fed with high fiber level; XL, Xiangcun pig group fed with low fiber level; XH, Xiangcun pig group fed with high fiber level; TL, Taoyuan pig group was fed with low fiber level; and TH, Taoyuan pig group was fed with high fiber level. Classification of degradation genes according to the type of microplastics (PET, polyethylene terephthalate; PE, polyethylene; PES, polyethylene succinate; PS, polystyrene; PHA, poly(3-hydroxyalkanoates); PHB, poly(3-hydroxybutyrate); PHO, poly(beta-hydroxyoctanoate); PLA, polylactic acid; PU, polyurethane; PVA, polyvinyl alcohol; TP, terephthalate; DP, diethyl phthalate; DEHP, di(2-ethylhexyl) phthalate; LDPE, low-density polyethylene; nylon: polyamide; PBAT, poly(butylene adipate-co-terephthalate); PBS, polybutylene succinate; PBSA, poly(butylene succinate-co-adipate); PCL, polycaprolactone).

**Figure 2:**
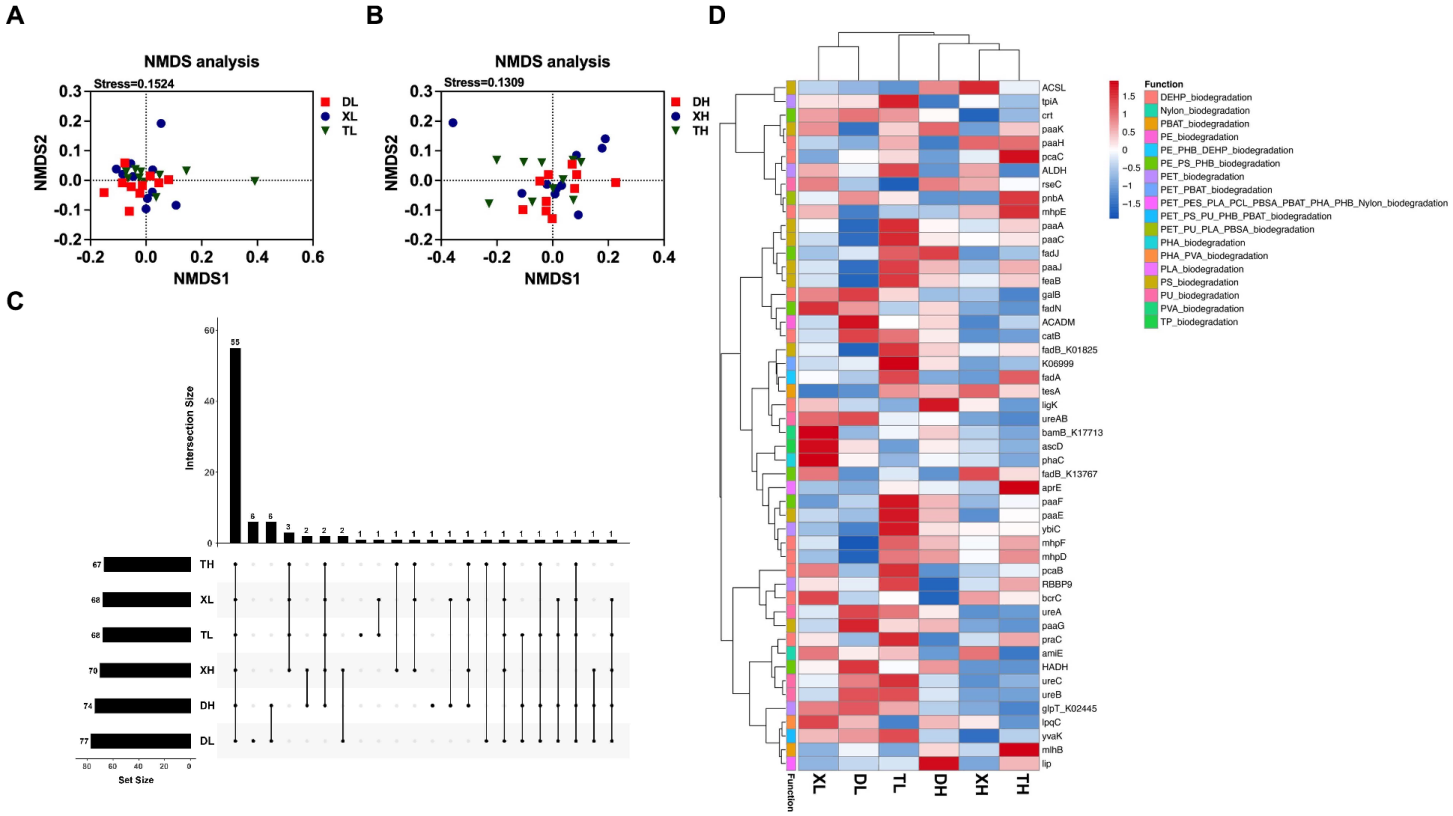
NMDS analysis of genes involved in the degradation of MPs in the colonic contents of different breeds of pigs at high and low fiber levels **(A,B)**. MP degradation genes upset plot **(C)**, the bars on the right side represent the number of MP degradation genes included in different groups, and the horizontal bars represent the number of MP degradation genes shared by different groups. Heatmap of enrichment analysis of the top 50 MP degradation genes with relative average abundance in different samples and groups **(D)**, different colors on the left side of the heatmap represent different MP degradation categories.

A total of 162 KOs on 18 MPs, including polyethylene (PE), polyethylene terephthalate (PET), poly(3-hydroxyalkanoates) (PHA), polystyrene (PS), and nylon (polyamide), and two plasticizers, namely, di(2-ethylhexyl) phthalate (DEHP) and dibutyl phthalate (DBP), were extracted from the results of the KEGG functional annotations. Marker gene set information, based on the RPKM abundance calculation method, was used for functional composition analysis and difference analysis. By classifying and statistically analyzing the different MP degradation types, it was determined that the main MP degradation types in the pig colon were PE, PET, and DEHP, but there was no difference in composition between diets with different fiber levels and between different breeds (Figure 1A). PCoA analysis (Figures 1B,C) showed that under a low-fiber diet, there was no difference in the genes for MP degradation in the colon between different pig breeds (Figures 1B,C). The similarity of intracolonic MP degradation genes between Duroc and Taoyuan pigs was relatively low under a high-fiber diet (Figure 1C). However, as a cross between Duroc and Taoyuan pigs, the MP degradation genes in the colons of Xiangcun pigs were a mixture of characteristics of the two parental breeds. In addition, we examined the α-diversity of MP degradation genes, and no differences were found in the Chao index (Figure 1D), and the Shannon index showed that a high-fiber diet significantly reduced the diversity of MP degradation genes in pig colons (Figure 1E). To overcome the shortcomings of the linear model (PCoA) and better reflect the non-linear structure, the accuracy of the model was assessed using NMDS stress values. We confirmed that the stress value of the Bray index was less than 0.2 (Figures 2A,B), which ensured the reliability of the model. The results showed that MP degradation genes in the colons of Duroc and Taoyuan pigs showed differences only under a high-fiber diet (Figure 2B) and that the MP degradation genes in the colons of Xiangcun pigs were a hybrid feature of the characteristics of the two parental breeds. Interestingly, the number of MP-degrading genes in the intestine of Duroc pigs was higher than that of Taoyuan and Xiangcun pigs, and both Duroc and Taoyuan pigs exhibited a reduction in the number of MP-degrading genes in the intestine after being fed a high-fiber diet (Figure 2C). Furthermore, we analyzed the MP degradation genes with an average abundance of the top 50 genes. Between breeds, the genes *praC* and *fadB_K13767* were significantly higher, and the genes *HADH* and *fadN* were significantly lower in the intestinal tract of Taoyuan pigs than in Duroc pigs. Between different fiber levels, a high-fiber diet significantly reduced the relative abundance of *tpiA, HADH, amiE, ureC, ureB, urea, paaG, ureAB, catB, fadN,* and *galB* (Figure 2D and Supplementary Table S1).

### Association of MP degradation genes with microorganisms in porcine colonic contents

**Figure 3:**
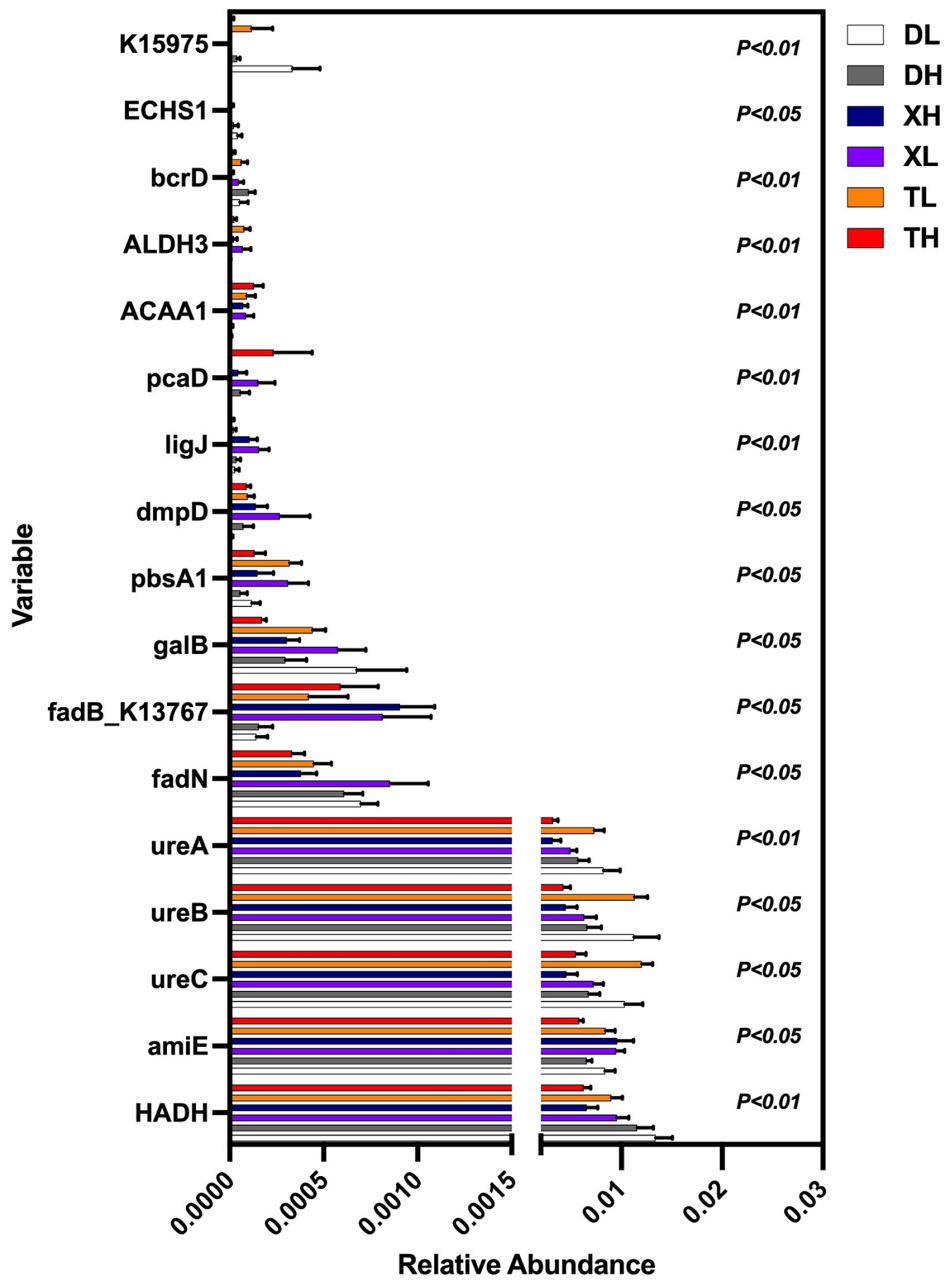
Multiple group comparisons of MP degradation genes in different groups.

**Figure 4:**
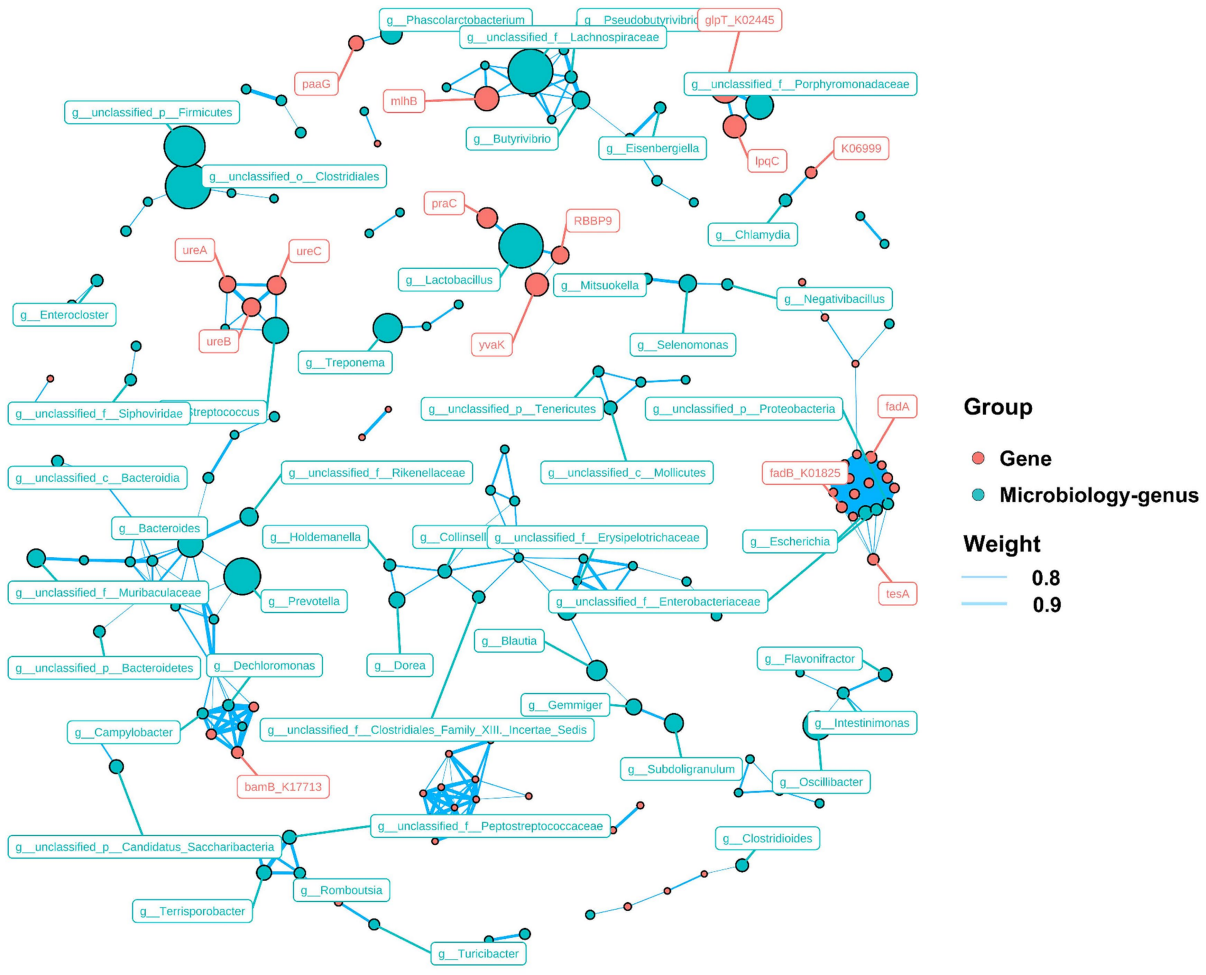
Correlation analysis of MP degradation genes and relative abundance of microorganisms.

**Figure 5:**
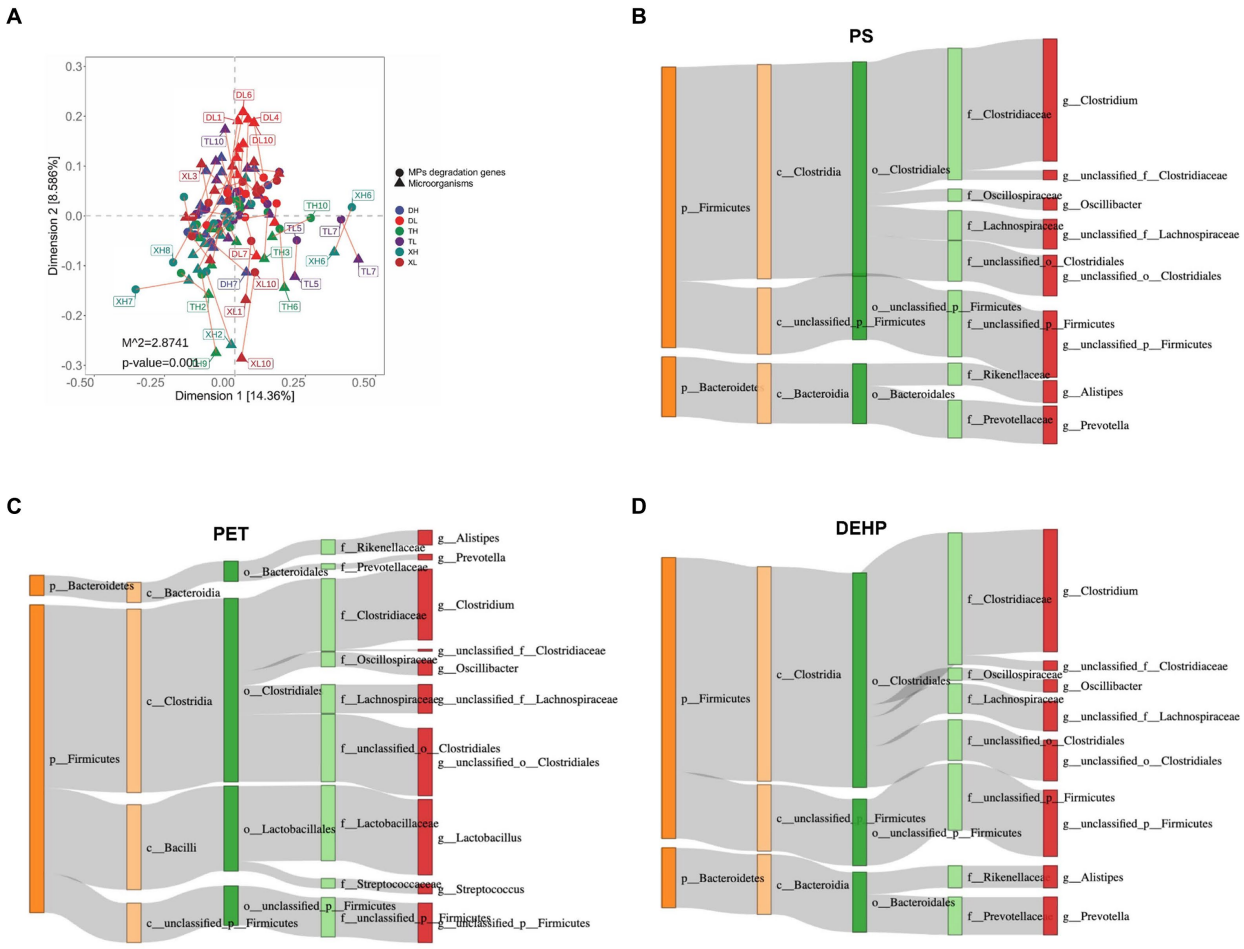
Associations between gut bacterial species and MP degradation gene abundance derived from Procrustes analyses **(A)**. Dots indicate the ranked position of MP degradation gene abundance in each sample, and triangles indicate the ranked position of bacterial species abundance. The length of the line between the dots and the triangles indicates the distribution of the Platts residual. PS **(B)**, PET **(C)**, and DEHP **(D)** degradation genes among the different categories of bacteria (unclassified microorganisms have been excluded from the analysis). The colors of the rectangles represent different classification levels. The length of the rectangles represents the number of MP degradation genes.

The non-parametric *Kruskal–Wallis* test confirmed significant differences (*p < 0.05*) among 17 MP degradation genes. *HADH* exhibited a relatively high abundance along with four other MP degradation genes and served as a biomarker (Figure 3). We performed Pearson correlation analysis of MP degradation gene profiles with genus-level microbes and selected highly significant and highly correlated (0.7 < |r| < 1, *p < 0.05*) relationships to demonstrate co-occurring networks, which indicated that microbes may be potential hosts for significantly enriched MP degradation gene profiles (Figure 4 and Supplementary Table S2). Procrustes analysis based on *Bray–Curtis* distance showed a good fit and significant correlation (M_2_ = 2.8741, *p = 0.001*) between the whole MP degradation gene profile and microbial community composition (Figure 5A). Furthermore, we performed microbial host analysis of the degradation genes of PS, PET, and DEHP, the main pollutants of MP pollution, and we found that the main host phylum-level microbes were *Firmicutes* and *Bacteroidetes*, and the main genus-level microbes were Clostridium. These results suggest that MP degradation gene profiles carried in colonic contents are consistent with the trend of their microbiomes, implying that differential MP degradation gene profiles may come from differential microorganisms (Figures 5B–D).

### Potential functions of MP degradation

To further investigate the enzymes involved in plastic biodegradation, we examined the results of KEGG functional annotation of known plastic-degrading enzymes reported in recent studies. A total of 72 putative degradation enzymes concerning the three main MP pollutants, namely, PS, PET, and DEHP, were identified, which were mainly classified as oxidoreductases, hydrolases, transferases, ligases, laccases, and isomerases (Supplementary Table S3). The functional modules associated with these enzymes show their involvement in reactions such as β-oxidation, benzoic acid degradation, and phenylacetic acid degradation (Supplementary Table S3).

**Figure 6:**
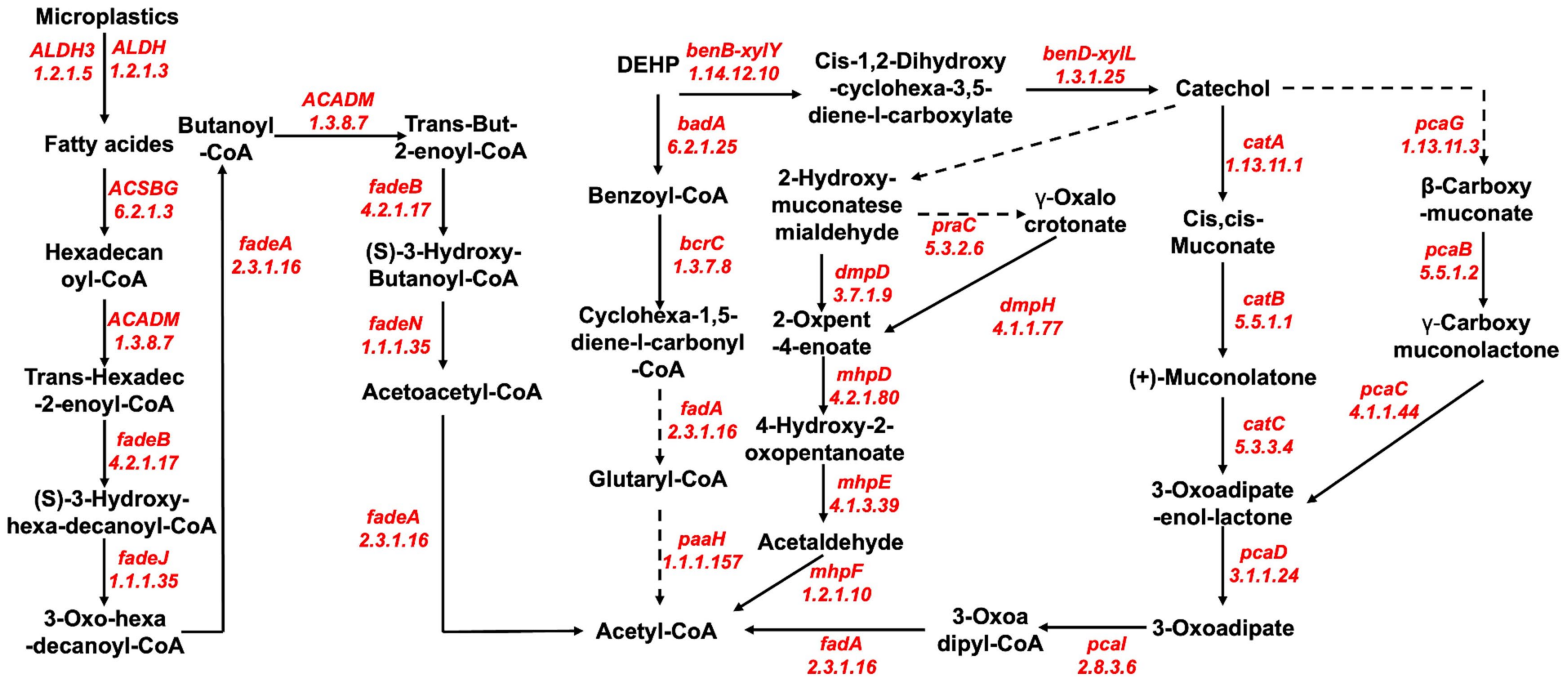
Reconstruction of putative pathways for bacterial degradation of microplastics (PS, PET, and DEHP) by enzymes and their involvement in metabolic pathways recovered from metagenomic data of porcine colonic content samples.

The main contaminants of MPs are MPs and plasticizers, but the degradation pathways of MPs and plasticizers show significant differences. The main microbial degradation pathway for MPs (PS and PET) begins with the conversion of MPs to fatty acids, which is mainly facilitated by enzymes such as aldehyde dehydrogenase (EC: 1.2.1.5 and EC: 1.2.1.3). Subsequently, a series of terminal or subterminal oxidations mediated by various enzymes, including ligases (EC: 6.2.1.3), oxidoreductases (EC: 1.3.8.7 and EC: 1.1.1.35), cleavage enzymes (EC: 4.2.1.17), and transferase enzymes (EC: 2.3.1.16), occur, yielding products that can be further metabolized by the beta-oxidation cycle (Figure 6).

The microbial degradation of DEHP is mainly via the benzoate metabolic pathway, which is divided into the benzoyl coenzyme A degradation pathway and the benzoate degradation pathway. The benzoyl coenzyme A degradation pathway is mainly characterized by the formation of benzoyl coenzyme A facilitated by ligases (EC: 6.2.1.5), followed by various enzyme-mediated degradation of benzoyl coenzyme A, mainly by oxidoreductases (EC: 1.3.7.8 and EC: 1.1.1.157) and transferase enzymes (EC: 2.3.1.16), and finally converted to acetyl coenzyme A. The benzoate degradation pathway first converts DEHP to catechol by oxidoreductase (EC: 1.14.12.10 and EC: 1.3.1.25) and catechol to acetyl coenzyme A by catechol ortho-cleavage and catechol meta-cleavage mode (Figure 6). Acetyl coenzyme A, a product of microbial degradation of MPs (PS and PET) and plasticizers (DEHP), is finally mineralized to CO_2_ and H_2_O after entering the citric acid cycle, acetate and dicarboxylate metabolism, or butyrate metabolism, representing the complete degradation of MPs.

## Discussion

MP pollution poses a serious threat to animals and ecosystems, and the degrading activities of microorganisms may help alleviate this pressure (Danso et al., 2019; Jain et al., 2023). Limited previous research has focused on the potential ability of porcine gut microbes to degrade MPs, and after MPs in feed and the environment are partially degraded by porcine gut microbes, their degradation products may be more readily broken down by other microorganisms in the soil, thus reducing the accumulation of MPs in the environment and the organism (Li et al., 2023; Wróbel et al., 2023). In this study, we analyzed potential degradation genes for MPs and their host microorganisms in samples of colonic contents from different breeds of pigs on different levels of fiber diet. Our results showed that porcine colons were enriched with genes related to MP degradation, with gut microbes as their main hosts, and that a fiber diet did not significantly affect the abundance of genes related to MP degradation, but significantly reduced their diversity. We thus hypothesized putative MP degradation pathways.

Degradation of MPs is mainly through physical abrasion, solar radiation, and biodegradation (Thakur et al., 2023). Due to the closed nature of the intestine, the primary route of MP removal from the gut is considered to be biodegradation (Wang et al., 2024b). A growing number of studies have shown that microorganisms have emerged as efficient MP degraders, producing enzymes associated with MP biodegradation, thereby significantly shortening the half-life of plastics (Yuan et al., 2020; Li et al., 2023). Through specific enzyme-mediated depolymerization and oxidative processes, the main backbone structure of plastics is converted into low molecular weight oligomers, which then undergo further aerobic mineralization to produce CO_2_ and H_2_O (Du et al., 2021; Sahu et al., 2023; Thakur et al., 2023). Based on the KEGG database and information from published literature, we identified the main MP and plasticizer degradation-related genes in porcine colonic contents. Similar to previous studies, we found that the most abundant MP-degrading gene carried by MP-degrading bacteria in the porcine intestine was ACSL, suggesting that the predominant mode of bacterial degradation of MPs is the same (Ou et al., 2024). Interestingly, we found that a high-fiber diet reduced the abundance of genes related to MP degradation. Microorganisms are considered to be a rich source of plastic-degrading enzymes and exhibit higher stability compared to plant and animal enzymes (Amobonye et al., 2021). Therefore, this may be because a high-fiber diet promotes the growth of probiotics and other beneficial bacteria, which may compete with MP degradation-associated bacteria, and thus reduce their abundance. It has been shown that MP-degrading bacteria are enriched in different polymers (Oberbeckmann and Labrenz, 2020; Zhai et al., 2023). In addition, MP degradation-associated genes drive changes in the composition of bacterial communities (Sun et al., 2021). Our results showed that the main genus-level microorganisms associated with MP degradation in the porcine intestine were Clostridium, which is similar to previous studies (Han et al., 2022; Lu et al., 2022; Wang et al., 2024a). Recent studies have also demonstrated that anaerobic digestion of MPs enriches Clostridium-like microorganisms during the process (Lu et al., 2022). Certainly, other bacteria, including these, are significantly correlated with MP degradation gene abundance, suggesting that the positive and negative correlation changes between these bacteria and function represent adjustments made by the gut flora to adapt to changes in the environment, which ultimately ensures functional redundancy of the gut flora. Synergistic interactions between various microorganisms enhanced the degradation kinetics of the plastic additives, facilitated the depolymerization of long polymer chains, and shortened the biodeterioration of the plastic particles (Liu et al., 2023).

According to current research on microbial plastic degradation, plastic polymer decomposition occurs primarily through the adsorption of enzymes to the polymer surface, and then the bonds are hydroperoxidized/hydrolyzed (Enyoh et al., 2022). Several studies have elucidated putative mechanisms associated with the degradation of MPs, involving hydroxylation and oxidation processes that produce carboxylic acids, which can be further catabolized and metabolized by bacteria through the β-oxidation cycle. However, the specific enzymes involved in these processes may vary depending on the type of MPs, bacterial species, and sample type (Yang et al., 2021). Based on MP-degrading enzymes recovered from porcine colon contents, we propose a potential mechanism for the microbial degradation of three major MP contaminants, namely, PS, PET, and DEHP. The overall degradation of PS and PET undergoes similar reactions to those previously described (Ou et al., 2024), including decomposition of the long polymer chains, oxidation of the short-chain oligomers, and ultimately complete mineralization (Chen et al., 2023). Although we have proposed a putative overall pathway for the degradation of MPs by porcine gut microbes and identified potential MP-degrading enzymes based on our analysis of the macrogenome, we still need to fully explore more detailed information on the degradation pathways, enzymes, and related intermediates of MPs to completely decipher the degradation mechanisms of more specific types of MPs.

## Conclusion

MP contamination is of increasing concern due to its persistence and harmful effects. We assessed MP degradation genes and associated bacterial communities in the colonic contents of different pig breeds on diets with different fiber levels. Our analyses of microbial functions confirmed that microbes are the main hosts of MP-degrading genes, with Lactobacillus spp. being the predominant host microorganisms. We also propose a putative degradation mechanism for three major MP pollutants, namely, PS, PET, and DEHP, based on the analysis of degradation enzymes. Our findings provide insights into the function of MP degradation genes and their host microorganisms in the porcine colon, suggesting a potential role for Lactobacillus species in MP biodegradation, as evidenced by their contribution to MP-degrading enzymes.

## Data availability statement

The datasets presented in this study can be found in online repositories. The names of the repository/repositories and accession number(s) can be found in the article/Supplementary material.

## Ethics statement

The animal study was approved by Institutional Animal Care and Use Committee of Sichuan Agricultural University, Sichuan, China (No. 20181105). The study was conducted in accordance with the local legislation and institutional requirements.

## Author contributions

TW: Conceptualization, Data curation, Formal analysis, Methodology, Validation, Visualization, Writing – original draft, Writing – review & editing. YL: Conceptualization, Data curation, Formal analysis, Methodology, Resources, Supervision, Writing – review & editing. BY: Conceptualization, Data curation, Formal analysis, Methodology, Project administration, Writing – review & editing. PZ: Conceptualization, Data curation, Formal analysis, Methodology, Project administration, Writing – review & editing. ZH: Conceptualization, Data curation, Formal analysis, Methodology, Project administration, Writing – review & editing. XM: Conceptualization, Data curation, Formal analysis, Methodology, Project administration, Writing – review & editing. JY: Conceptualization, Data curation, Formal analysis, Methodology, Project administration, Writing – review & editing. JL: Conceptualization, Data curation, Formal analysis, Methodology, Project administration, Writing – review & editing. HY: Conceptualization, Data curation, Formal analysis, Methodology, Project administration, Writing – review & editing. JH: Conceptualization, Data curation, Formal analysis, Funding acquisition, Resources, Validation, Visualization, Writing – original draft, Writing – review & editing.

## Footnotes

^1^ https://cran.r-project.org/web/packages/networkD3
